# University students’ perceptions of social media use and intellectual security: a cross-sectional study in Egypt

**DOI:** 10.3389/fsoc.2026.1877494

**Published:** 2026-07-23

**Authors:** Zezit M. Noufal, Eman Ahmed Mohamed Ali, Marzouqah Alazmi, Alaa Al-Taii, Kayaty Ashour

**Affiliations:** 1 College of Arts, Humanities and Social Sciences, University of Khorfakkan, Khorfakkan, Sharjah, United Arab Emirates; 2 Department of Sociology and Social Work, College of Social Sciences, Kuwait University, Safat, Kuwait; 3 Department of Sociology, College of Arts, Humanities and Social Sciences, University of Sharjah, Sharjah, United Arab Emirates; 4 Department of Sociology, Faculty of Arts, Beni-Suef University, Beni-Suef, Egypt

**Keywords:** social media, intellectual security, university students, media institutions, digital literacy, counter-extremism, extremism, Egypt

## Abstract

**Introduction:**

Intellectual security—understood as critical awareness, balanced judgment, and resilience to manipulative or extremist persuasion—is shaped within digital environments. This study examined university students’ perceptions of social media and of media institutions’ role in countering extremist discourse.

**Methods:**

A cross-sectional survey was conducted with 384 undergraduates at Port Said University, Egypt, recruited through non-probability convenience sampling. The researcher-developed questionnaire included demographic and platform-use items, a 20-item scale assessing the perceived awareness-oriented role of social media in intellectual security, and a 5-item scale assessing the perceived institutional-media role in countering extremism. Pilot validation involved 164 cases. Analyses included reliability testing, principal-axis factoring, descriptive statistics, Pearson correlations, multiple linear regression, and model diagnostics.

**Results:**

Internal consistency was *α* = 0.922 for the awareness-oriented scale and α = 0.802 for the institutional-media scale. Students reported high endorsement of both roles (M = 3.96, SD = 0.42; M = 4.03, SD = 0.39), and the scales were positively correlated (r = 0.615). In adjusted models, daily social media use was the strongest predictor of both outcomes (β = 0.503 and β = 0.585, respectively; *p* < 0.001). Facebook use retained smaller positive associations (β = 0.173, *p* < 0.001; β = 0.109, *p* = 0.026); adjusted R² values were 0.500 and 0.412.

**Discussion:**

Students perceived intellectual security as linked to digital engagement, critical interpretation, and institutional communication. These findings reflect associations rather than causal platform effects, direct exposure to extremist content, or demonstrated institutional effectiveness.

## 1 Introduction

Digital communication technologies have reshaped how young people access information, form opinions, and participate in public discourse. Among these technologies, social media platforms are especially influential because they combine immediacy, interactivity, algorithmic curation, and wide social reach. For university students, these platforms are not only spaces for communication and entertainment; they are also environments in which political, religious, and moral ideas circulate, gain visibility, and compete for legitimacy (Aichner et al., 2021; Thorson, 2020).

Within Arab intellectual-security scholarship, the concept has commonly been discussed as a preventive sociocultural condition that protects young people from ideological distortion, extremism, and cognitive vulnerability while preserving balanced judgment and social responsibility. Al-Dajah (2019) frames intellectual security as a contemporary requirement for maintaining social cohesion and protecting individuals from destabilizing ideas, while Al-Jedaiah (2021) links intellectual security directly to broader efforts to counter extremism and terrorism. Recent field-based work has also emphasized that intellectual security among university students is shaped by educational, cultural, social, and institutional factors rather than by individual media exposure alone (Al-Halalat et al., 2024). This body of work supports the present study’s treatment of intellectual security as a multidimensional construct involving awareness, evaluative judgment, institutional mediation, and resilience against manipulative discourse.

The role of social media in intellectual formation is inherently paradoxical. On the one hand, these platforms can support awareness, moderation, and resilience by enabling access to educational content, official guidance, responsible digital citizenship practices, and constructive public discussion (Ahmed and Mohamed Gad, 2019; Al-Amer et al., 2023; Alsubaie et al., 2026; Mekheimer and Abdelhalim, 2025).

On the other hand, the same platforms can intensify exposure to misinformation, ideological polarization, and extremist narratives (Ahmed and Mohamed Gad, 2019; Al-Amer et al., 2023; Hollewell and Longpré, 2022). The issue is therefore not whether social media is inherently protective or inherently harmful, but how students interpret what they encounter and whether institutions help shape that interpretation.

Youth engagement with social media must also be understood as part of broader networked youth culture. Boyd (2014) argues that social media platforms function as socially embedded environments in which young people construct identity, negotiate belonging, and encounter public issues through peer-mediated interaction. From this perspective, students’ perceptions of intellectual security are unlikely to be shaped only by formal institutional messaging; they also emerge through everyday platform participation, peer circulation, and repeated exposure to socially endorsed content. This supports the present study’s inclusion of daily-use intensity and platform participation as indicators of students’ perceived digital environment.

The present study adopts a perceptual and interpretive analytic orientation rather than an objective effects framework. Its empirical focus is not on determining whether social media directly attenuates extremist tendencies, produces measurable gains in intellectual security, or enhances the operational effectiveness of media institutions. This distinction is important because digital communication environments are shaped by platform affordances, algorithmic visibility, participatory circulation, and institutional moderation, but these structural conditions do not by themselves establish measurable behavioral outcomes (Gillespie, 2018; Thorson, 2020; van Dijck et al., 2018).

Instead, the study investigates how university students construe the awareness-building, moderating, and institutionally mediated functions of social media within their routine digital ecologies, particularly in relation to media literacy, evaluative judgment, misinformation recognition, and resilience against manipulative or extremist discourse (Buckingham, 2015; Livingstone, 2004; Wardle and Derakhshan, 2017). Accordingly, the findings are interpreted as evidence of perceived roles, perceived institutional relevance, and self-reported evaluative orientations toward digital communication, rather than as causal indicators of behavioral change, platform impact, or institutional performance in countering extremism (Conway, 2017; Hollewell and Longpré, 2022; Mitts, 2025).

In this study, intellectual security is defined operationally as cognitive resilience against manipulative or extremist persuasion, critical awareness, and the capacity for balanced judgment in contested information environments. This definition is important for an international readership because the concept is sometimes misread as a synonym for ideological control. Here, it is used in a preventive and educational sense: not the suppression of thought, but the strengthening of reflective judgment, discernment, and resistance to manipulation. Conceptually, this use of intellectual security aligns with Arab scholarship that treats the construct as an educational and preventive condition concerned with protecting critical judgment from extremist, exclusionary, or manipulative persuasion rather than limiting legitimate diversity of thought (Al-Dajah, 2019; Al-Jedaiah, 2021). Recent field evidence further suggests that university students’ intellectual security is shaped by multiple interacting factors, including educational climate, institutional guidance, value formation, and exposure to digital discourses (Al-Halalat et al., 2024).

This educational interpretation is consistent with recent regional work that treats intellectual security as a dimension of responsible digital citizenship, ethical online participation, and the cultivation of students’ capacity to act freely but responsibly in digital public spaces (Alsubaie et al., 2026; Mekheimer and Abdelhalim, 2025).

This distinction also clarifies the relationship between protection and freedom. Intellectual security, as used in the present study, does not imply imposing a single viewpoint or restricting legitimate intellectual diversity. Rather, it refers to building the critical capacities that allow students to engage with competing ideas without becoming vulnerable to deceptive, absolutist, or extremist persuasion.

In this sense, intellectual security is compatible with freedom of thought because it depends on critical literacy, ethical online responsibility, and reflective public participation rather than enforced conformity (Mekheimer and Abdelhalim, 2025).

The international literature on digital media helps clarify why this distinction matters. Platformed public life is shaped by algorithmic visibility, participatory circulation, peer endorsement, and institutional content moderation (Gillespie, 2018; van Dijck et al., 2018). Digital-literacy scholarship similarly emphasizes that users need interpretive skills to evaluate claims, recognize misinformation, and distinguish persuasive content from manipulative or extremist messaging (Buckingham, 2015; Livingstone, 2004; Wardle and Derakhshan, 2017). Research on online radicalization further shows that extremist persuasion often develops through repeated exposure, network reinforcement, and platform-specific affordances rather than through one-directional propaganda alone (Conway, 2017; Hollewell and Longpré, 2022; Mitts, 2025).

Research on violent extremism and counter-extremism also demonstrates that digital platforms can be used both to circulate extremist narratives and to support preventive communication. Sageman (2008) emphasized the decentralized and networked character of contemporary extremist mobilization, while Rabasa et al. (2010) highlighted the importance of deradicalization and disengagement strategies that address ideological, social, and communicative dimensions. More recent work has extended this concern to social media, showing that countering violent extremism requires platform-aware communication strategies that can respond to online circulation, audience segmentation, and the social visibility of extremist or counter-extremist messages (Amit et al., 2021). These studies reinforce the need to examine how students perceive institutional and media actors as participants in counter-extremism communication.

Related educational research in Arab and Egyptian contexts also shows that digital literacy, AI literacy, and digital critical thinking are increasingly treated as forms of civic and cultural competence rather than merely technical skills (Mekheimer and Tohamy, 2026).

Existing Arab scholarship confirms the relevance of this issue but also reveals important gaps. Much of the available work is cross-sectional and descriptive; many studies emphasize the risks of extremist dissemination without equally examining awareness-building functions; and fewer studies incorporate students’ perceptions of media institutions within the same analytical design. Studies from Arab university contexts further show that social media is often perceived as both a resource for awareness and a potential threat to intellectual security. Al-Khataibeh (2019), for example, examined social-media extremism as a perceived intellectual-security threat among university students, showing that digital environments may expose youth to ideological content that requires critical filtering and institutional guidance. In Egypt, Amin (2024) similarly found that Facebook can shape perceptions of intellectual security, highlighting the continued importance of platform-specific analysis in Egyptian sociocultural contexts. These studies justify examining both general social media use and specific platform indicators when studying students’ perceptions of intellectual security.

The study contributes to the literature by integrating students’ perceptions of social media, platform-use intensity, and the perceived institutional-media role in countering extremist discourse within a single empirical model in an Egyptian university context. These gaps are especially visible in Egyptian university settings, where empirical work on intellectual security in digital environments remains limited. The present study addresses this gap by examining both the awareness-oriented and institutional dimensions of social media within a single dataset from Port Said University.

Accordingly, this study asks how students at Port Said University perceive social media as a possible environment for awareness, moderation, and intellectual resilience, and how they perceive media institutions as potential actors in countering extremist discourse. It also examines whether daily-use intensity and platform-use patterns are associated with stronger endorsement of these perceived roles.

### 1.1 Research questions

1. To what extent do university students perceive social media as contributing to awareness, moderation, and intellectual security?
2. To what extent do students perceive media institutions as using social media to counter extremist ideologies and support intellectual security?
3. How are Axis 1 and Axis 2 associated with daily-use intensity and platform-use patterns?
4. Which usage-related and background variables significantly predict awareness-oriented intellectual security perceptions and endorsement of the institutional-media role in countering extremism?

### 1.2 Hypotheses

1. Students’ perceptions of the awareness-oriented role of social media in promoting intellectual security are positively associated with their perceptions of the institutional-media role in countering extremist discourse.
2. Higher daily social media use positively predicts stronger perceptions of social media as a means of promoting awareness, moderation, and intellectual security.
3. Daily social media use and selected platform-use patterns are positively associated with stronger perceptions of the institutional-media role in countering extremism and supporting intellectual security.

## 2 Literature review and theoretical framework

Recent scholarship has increasingly emphasized the dual role of social media in relation to youth intellectual security. Ahmed and Mohamed Gad (2019) showed that social networking sites can contribute to intellectual awareness when used as channels for guidance and moderated public engagement. Al-Amer et al. (2023) likewise found that university students may view social media as useful in confronting intellectual extremism when institutional messaging is credible and sustained. At the same time, research on online radicalization indicates that networked repetition, emotional amplification, and peer reinforcement can make digital environments important in the spread of extremist narratives (Ammar and Xu, 2018; Conway, 2017; Hollewell and Longpré, 2022; Mitts, 2025).

Comparable regional research has shown that social media may function as an intellectual-security risk when extremist ideas circulate among university undergraduates through repeated exposure, peer sharing, and persuasive digital framing (Al-Khataibeh, 2019). Egyptian evidence on Facebook also suggests that platform-specific practices can shape users’ perceptions of intellectual security, particularly where public discussion, moral evaluation, and awareness-oriented messaging intersect (Amin, 2024).

The literature review broadens the study beyond local risk discourse by drawing on four international strands: media literacy, platform affordances, digital public spheres, and radicalization research. Media-literacy scholarship emphasizes the interpretive capacities required to evaluate digital information, assess credibility, recognize manipulation, and participate responsibly in networked communication (Buckingham, 2015; Livingstone, 2004). Platform studies show that visibility and circulation are not neutral; they are shaped by platform design, moderation systems, recommender logics, and the publicness or privateness of communication environments (Gillespie, 2018; Thorson, 2020; van Dijck et al., 2018).

Digital youth studies further show that young people’s online lives are shaped by peer networks, visibility, identity work, and platform norms, making intellectual security inseparable from everyday digital participation (Boyd, 2014). Recent higher-education research likewise connects digital citizenship, ethical online behavior, AI literacy, and digital critical thinking with students’ capacity to participate responsibly in networked environments (Alshehri et al., 2026; Alsubaie et al., 2026; Mekheimer and Abdelhalim, 2025; Mekheimer and Tohamy, 2026).

Studies of misinformation, networked polarization, and digital threat construction are also relevant to the present study. False or manipulative content may be amplified through selective exposure, peer sharing, and algorithmically reinforced attention patterns, thereby shaping how users interpret public issues and evaluate social risk (Cinelli et al., 2021; Guess et al., 2019; Wardle and Derakhshan, 2017). In the Egyptian context, recent work on social media and threat construction shows that digital platforms can contribute to the circulation of exclusionary narratives and socially constructed perceptions of danger, particularly when public issues are framed through fear, burden, or symbolic threat (Ashour et al., 2026). Although that study focuses on refugee-related discourse rather than intellectual security directly, it is relevant here because it demonstrates how Egyptian social media can transform contested public issues into emotionally charged narratives. These dynamics are not identical to violent extremism, but they help explain why cognitive resilience, critical literacy, institutional correction, and balanced evaluative judgment are central to students’ perceptions of intellectual security in digital environments (Buckingham, 2015; Livingstone, 2004; Rabasa et al., 2010; Sageman, 2008).

**Figure 1:**
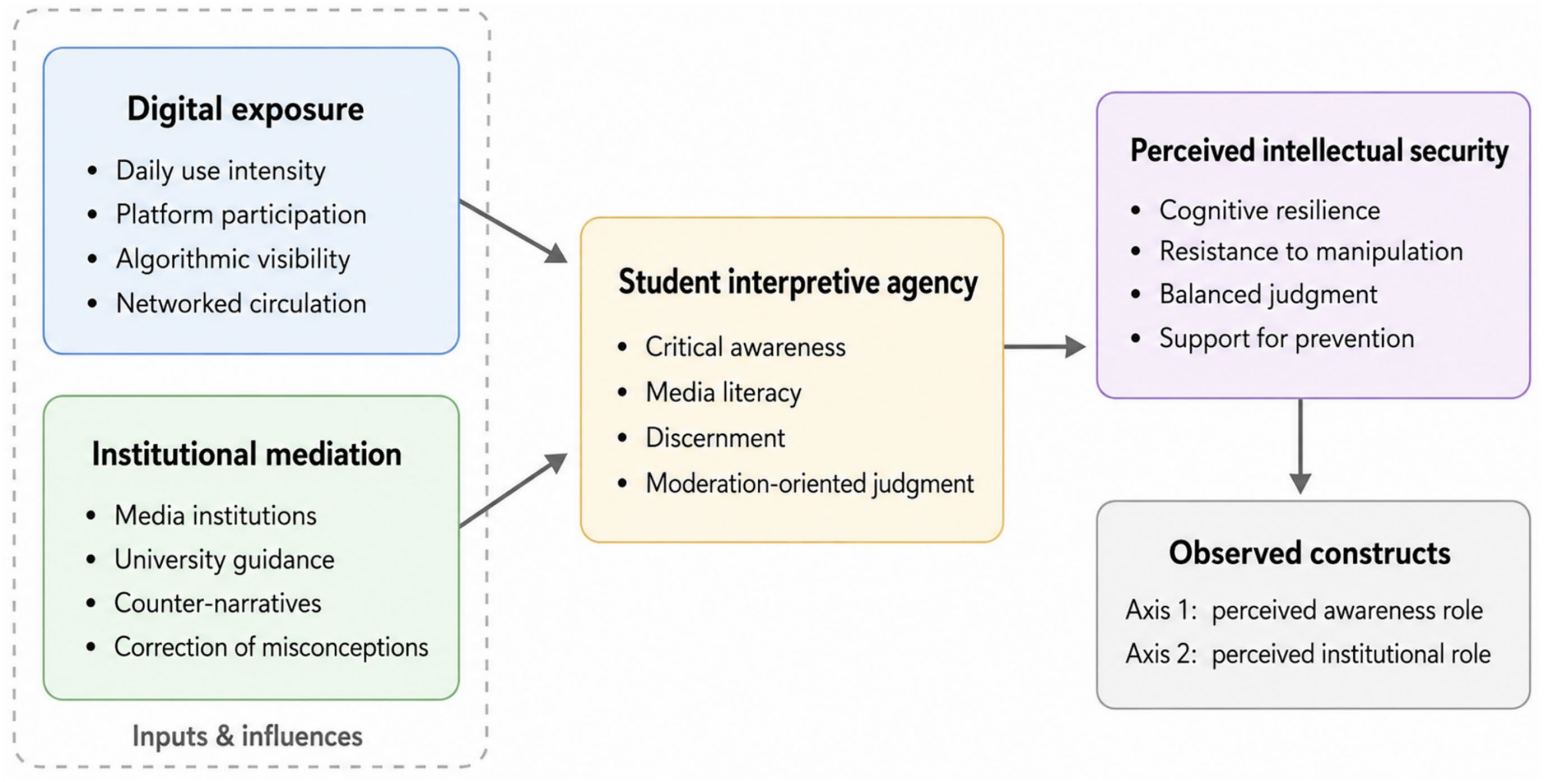
Digital-social ecology of intellectual security (DSEIS) model.

To interpret these relationships, the study uses the Digital-Social Ecology of Intellectual Security (DSEIS) as a study-specific analytical lens rather than as a fully tested causal framework (Figure 1). The model assumes that intellectual security is shaped through the interaction of digital exposure, individual interpretive agency, institutional mediation, and perceived outcomes. In the present study, digital exposure is represented by daily-use intensity and platform-use patterns; interpretive agency is reflected indirectly through Axis 1; institutional mediation is represented by Axis 2; and the outcome dimension is treated as perception-based endorsement rather than observed behavioral change.

However, these studies tend to address intellectual security, digital citizenship, social-media risk, AI literacy, or extremist communication as separate domains. Fewer studies combine awareness-oriented intellectual security, institutional media roles, and platform-use patterns within one empirical design. The present study addresses this gap by examining how Egyptian university students perceive both the protective role of social media and the institutional-media role in countering extremist discourse.

This framework prevents reductionist interpretation. Social media does not simply produce moderation or extremism by itself. Its perceived relationship with intellectual security depends on how frequently students engage with platforms, what forms of content become visible, whether institutions intervene credibly, and whether users possess critical capacities for evaluating contested messages. For that reason, DSEIS connects the study variables to a broader sociological understanding of youth media culture without claiming that the present cross-sectional dataset can test a full causal ecology.

Considered together, this literature suggests that intellectual security in digital environments cannot be reduced to either individual belief or institutional control. It is shaped by the interaction of platform affordances, youth digital culture, perceived ideological risk, and the credibility of institutional counter-narratives (Al-Dajah, 2019; Al-Jedaiah, 2021; Amit et al., 2021; Boyd, 2014; Sageman, 2008). The present study therefore examines students’ perceptions of social media use and institutional-media engagement within a single empirical model, while treating the results as perception-based associations rather than evidence of direct behavioral or institutional effects.

## 3 Method

### 3.1 Research design

The study employed a descriptive-analytical cross-sectional survey design to examine students’ perceptions of the relationship between social media use and intellectual security and their perceptions of the role of media institutions in countering extremist ideologies. Because all variables were collected at one time point through self-report, the design supports descriptive and associational inference only.

### 3.2 Setting, recruitment, and sample

The target population consisted of undergraduate students enrolled at Port Said University in Egypt. Participants were recruited through university-related digital communication channels and departmental student groups. The study used a non-probability convenience sampling design because participants were approached through accessible student networks rather than through a probability-based sampling frame. Students were eligible to participate if they were enrolled as undergraduates, fell within the age categories used in the questionnaire, used social media, and agreed voluntarily to complete the survey. Questionnaires with incomplete demographic information or missing responses on the substantive scale items were excluded through listwise screening. The final valid sample consisted of 384 complete questionnaires. Because recruitment was conducted through decentralized faculty and student communication channels, the exact number of students who received the survey invitation could not be reliably established; therefore, a formal response rate is not reported. This limitation is acknowledged when interpreting the generalizability of the findings.

### 3.3 Instrument

Data were collected through a researcher-developed questionnaire composed of demographic and platform-use items and two substantive axes. Demographic variables captured gender, age group, faculty, academic level, average daily social media use, and regular use of WhatsApp, Facebook, Instagram, Twitter/X, and Snapchat. The cleaned analytic coding file treated daily social media use as five ordered categories: less than 1 h, about 1 h, 1–3 h, 4–6 h, and more than 6 h. Platform indicators were coded 0 = no and 1 = yes.

Axis 1 comprised 20 items measuring students’ perceptions of the role of social media in enhancing awareness, moderation, and intellectual security. Axis 2 comprised 5 items measuring students’ perceptions of the role of media institutions and digital media in countering extremism and supporting intellectual security. All substantive items were rated on a five-point Likert scale ranging from 1 (strongly disagree) to 5 (strongly agree), with higher scores indicating stronger endorsement of the relevant perceived role. The full item list is provided in Appendix A (Supplementary Table 2) to support replication.

### 3.4 Content validity, pilot validation, and reliability

The questionnaire was researcher-developed and designed to measure two related perceptual dimensions: students’ perceptions of the awareness-oriented role of social media in supporting intellectual security and their perceptions of the institutional-media role in countering extremist discourse. The item pool was developed from the objectives of the study and informed by prior scholarship on intellectual security, social media use, digital literacy, and counter-extremism communication. In particular, item development was guided by conceptualizations of intellectual security as a preventive educational construct (Al-Dajah, 2019; Al-Jedaiah, 2021), university-based evidence on factors shaping intellectual security (Al-Halalat et al., 2024), studies of social-media risk among university youth (Al-Khataibeh, 2019), Facebook-specific evidence from Egypt (Amin, 2024), and higher-education research on digital citizenship and ethical online behavior (Alsubaie et al., 2026; Mekheimer and Abdelhalim, 2025).

Content validity was established through an iterative qualitative review process. A panel of experts in sociology and media studies evaluated the item pool for linguistic clarity, cultural appropriateness, conceptual relevance, and alignment with the study’s operational definition of intellectual security. Items were revised following this review until consensus was reached on face and content validity. Because the retained audit materials do not preserve the experts’ individual ratings, numerical content-validity index values, such as I-CVI or S-CVI, were not aggregated and are therefore not reported.

A pilot study (*n* = 164) was conducted prior to the main data collection to refine the instrument and assess the statistical performance of the two scales. This phase focused on reliability, factorability, and item clarity. Procedural feedback from pilot participants was used to finalize the wording of the Axis 1 and Axis 2 items. Because the pilot was intended for internal validation, detailed recruitment logs for this subsample were not retained in the final audit materials.

The pilot data provided supportive quantitative evidence for the instrument. Reliability analysis showed acceptable internal consistency for Axis 1 (Cronbach’s *α* = 0.922, 20 items) and Axis 2 (Cronbach’s α = 0.802, 5 items). Principal-axis factoring was used as an exploratory validation procedure because the questionnaire was developed for the Egyptian university context rather than adopted from a previously confirmed scale. Axis 1 showed excellent sampling adequacy (KMO = 0.936) and a significant Bartlett test, χ^2^(190) = 1258.86, *p* < 0.001; three factors with eigenvalues greater than 1 were extracted, accounting for 42.51% of the extracted variance after rotation. Axis 2 showed good sampling adequacy (KMO = 0.829) and a significant Bartlett test, χ^2^ (10) = 231.13, *p* < 0.001; one factor was extracted, with an initial eigenvalue of 2.80 and extracted variance of 45.50%. These results support preliminary construct validity, although future multi-site studies should use confirmatory factor analysis to test whether the factor structure is replicated across broader populations.

### 3.5 Ethics statement

The survey was anonymous, non-interventional, and minimal risk. No personally identifying information was collected or retained. Participants were informed of the study purpose, the voluntary nature of participation, and the anonymous handling of responses before completing the questionnaire. Proceeding with the questionnaire was treated as consent to participate. Because the study involved anonymous self-report survey data and did not include clinical, experimental, invasive, or identifiable human-subject procedures, written informed consent was not required. No animal studies or identifiable human data are included.

### 3.6 Statistical analysis

**Figure 2:**
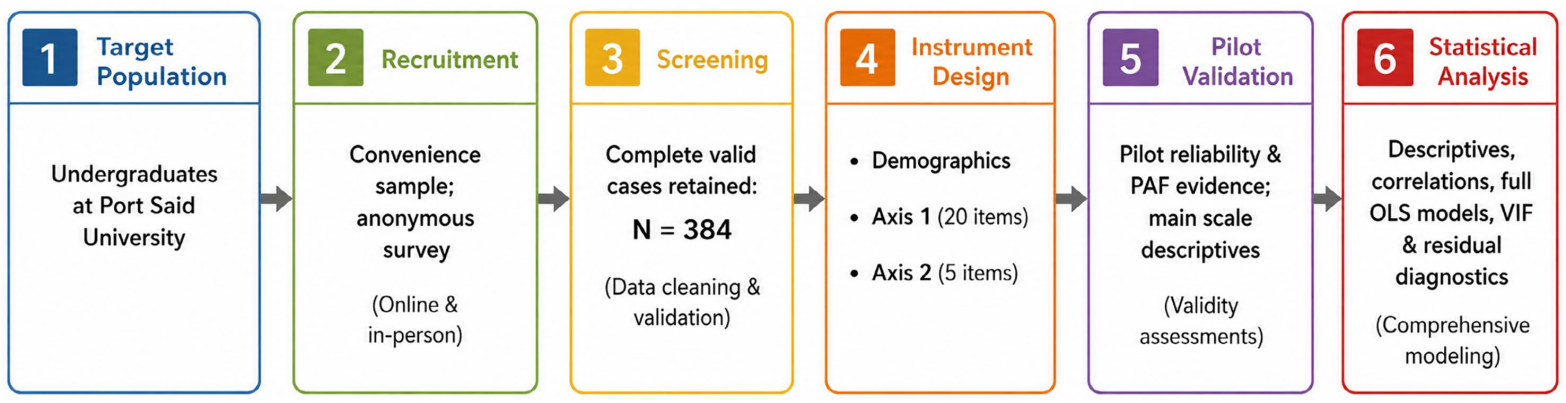
Study design and analytical workflow.

The overall methodological workflow, from target population identification to statistical modeling, is summarized in Figure 2. All statistical analyses were conducted using IBM SPSS Statistics version 22.0. The outputs were subsequently reviewed through internal consistency checks to confirm alignment among variable coding, descriptive summaries, and model-based estimates.

Descriptive statistics summarized demographic characteristics, platform-use patterns, and scale-level responses. Pearson correlations examined relationships among Axis 1, Axis 2, daily-use intensity, and cumulative platform count. Because daily-use intensity and platform count were highly correlated (*r* = 0.849), platform count was reported descriptively and correlationally but was not entered into the regression models together with daily use. This decision reduced avoidable multicollinearity while retaining specific platform-use indicators.

Two full multiple linear regression models were estimated. Axis 1 and Axis 2 were separately regressed on daily-use intensity, gender, age, faculty, academic level, and specific platform-use indicators. Gender was coded 1 = male and 0 = female. Age was entered as an ordered category. Faculty was represented by dummy variables for law, science, commerce, and other, with arts as the reference category. Academic level was represented by dummy variables for second, third, and fourth year or above, with first year as the reference category. Daily use was entered as an ordered intensity variable. Platform use was coded 1 = regular use and 0 = non-use. Regression tables report unstandardized coefficients, standard errors, standardized coefficients, 95% confidence intervals, *p* values, tolerance, and VIF.

The robustness of the regression models was evaluated through post-estimation diagnostic procedures, as indicated in the final stage of the workflow in Figure 2. Multicollinearity was assessed using tolerance statistics and variance inflation factors, while residual independence was examined through the Durbin–Watson statistic. Model specification and error variance assumptions were further evaluated using Breusch–Pagan tests for heteroscedasticity, inspection of standardized residual ranges, and Cook’s distance for influential observations. These diagnostic checks were used to assess the stability and interpretability of the estimated coefficients. Given the cross-sectional and self-report nature of the study, the resulting estimates are treated as associational patterns in students’ perceptions rather than as evidence of causal effects.

## 4 Results

The results are presented in six parts: descriptive sample and platform-use profile, preliminary scale validation and descriptives, correlations among the principal variables, full regression model for Axis 1, full regression model for Axis 2, and regression diagnostics.

### 4.1 Descriptive profile of the sample and platform use

Table 1 summarizes the demographic composition of the final valid sample and platform-use patterns. The sample was balanced by gender and concentrated mainly in the 17–23 age range. WhatsApp and Facebook were the most frequently reported platforms. Daily social media use was distributed across five ordered categories in the cleaned dataset.

**Table 1:** Participant characteristics and platform use (*N* = 384).

Variable	Category	*n* (%)
Gender	Male	196 (51.0)
	Female	188 (49.0)
Age group	17–20 years	150 (39.1)
	21–23 years	170 (44.3)
	24–26 years	64 (16.7)
Faculty	Arts	70 (18.2)
	Law	60 (15.6)
	Science	130 (33.9)
	Commerce	74 (19.3)
	Other	50 (13.0)
Academic level	First year	60 (15.6)
	Second year	90 (23.4)
	Third year	84 (21.9)
	Fourth year or above	150 (39.1)
Daily use	Less than 1 h	30 (7.8)
	About 1 h	50 (13.0)
	1–3 h	120 (31.3)
	4–6 h	110 (28.6)
	More than 6 h	74 (19.3)
Platform use	WhatsApp	288 (75.0)
	Facebook	273 (71.1)
	Instagram	161 (41.9)
	Twitter/X	88 (22.9)
	Snapchat	92 (24.0)

Percentages are based on the full sample (*N* = 384). Platform-use items allowed multiple responses; therefore, platform percentages do not sum to 100%.

### 4.2 Scale validation, reliability, and descriptive statistics

Table 2 reports the pilot validation evidence and final scale descriptives. Both axes recorded high mean scores in the final dataset, indicating strong endorsement of the perceived awareness-oriented role of social media and the perceived institutional-media role in countering extremism.

**Table 2:** Preliminary scale validation and final descriptive statistics.

Scale	Pilot *n*	Items	α	KMO	Bartlett’s test	PAF solution	Final *M* (*SD*)
Axis 1: Social media role in intellectual security	164	20	0.922	0.936	χ^2^ (190) = 1258.86, *p* < 0.001	Three factors; 42.51% variance	3.96 (0.42)
Axis 2: Institutional-media role in countering extremism	164	5	0.802	0.829	χ^2^ (10) = 231.13, *p* < 0.001	One factor; 45.50% variance	4.03 (0.39)

PAF, principal-axis factoring. Pilot results provide preliminary construct evidence. Final descriptive statistics are based on the final sample (*N* = 384).

### 4.3 Correlational structure

Table 3 shows positive associations among the principal variables. Axis 1 was positively associated with Axis 2 (r = 0.615), daily-use intensity (r = 0.702), and platform count (r = 0.657). Axis 2 was also positively associated with daily-use intensity (r = 0.643) and platform count (r = 0.564). The high correlation between daily use and platform count (r = 0.849) justified excluding platform count from the regression models to avoid redundant predictors.

**Table 3:** Correlations among key variables.

Variable	Axis 1	Axis 2	Daily use	Platform count
Axis 1	1.000	0.615	0.702	0.657
Axis 2	0.615	1.000	0.643	0.564
Daily use	0.702	0.643	1.000	0.849
Platform count	0.657	0.564	0.849	1.000

All correlations are Pearson correlations and were significant at *p* < 0.001.

### 4.4 Multiple regression predicting Axis 1

The full model predicting Axis 1 was statistically significant, R = 0.721, R^2^ = 0.520, adjusted R^2^ = 0.500, *F* (15, 368) = 26.58, *p* < 0.001. Daily-use intensity was the strongest positive predictor (B = 0.183, *β* = 0.503, *p* < 0.001). Facebook use also retained a smaller positive association (B = 0.161, β = 0.173, *p* < 0.001). The remaining background and platform variables were not statistically significant after all predictors were entered (Table 4).

**Table 4:** Full multiple regression model predicting Axis 1.

Predictor	B	SE	β	t	*p*	95% CI for B	Tolerance	VIF
Daily use	0.183	0.025	0.503	7.19	<0.001	0.133, 0.233	0.266	3.76
Gender: male	0.001	0.031	0.001	0.04	0.970	−0.060, 0.063	0.953	1.05
Age	−0.010	0.022	−0.016	−0.44	0.663	−0.054, 0.034	0.918	1.09
Faculty: law	−0.013	0.053	−0.011	−0.24	0.811	−0.117, 0.092	0.626	1.60
Faculty: science	−0.028	0.045	−0.031	−0.62	0.538	−0.115, 0.060	0.520	1.92
Faculty: commerce	0.004	0.051	0.003	0.07	0.942	−0.096, 0.103	0.584	1.71
Faculty: other	−0.046	0.056	−0.037	−0.83	0.407	−0.156, 0.064	0.656	1.52
Level: second	0.008	0.050	0.008	0.16	0.871	−0.090, 0.107	0.515	1.94
Level: third	−0.010	0.051	−0.010	−0.21	0.838	−0.110, 0.090	0.525	1.90
Level: fourth or above	−0.006	0.046	−0.007	−0.13	0.893	−0.097, 0.085	0.454	2.20
WhatsApp	0.075	0.047	0.077	1.60	0.111	−0.017, 0.166	0.569	1.76
Facebook	0.161	0.042	0.173	3.83	<0.001	0.078, 0.243	0.644	1.55
Instagram	0.005	0.040	0.006	0.13	0.893	−0.073, 0.084	0.593	1.69
Twitter/X	0.021	0.042	0.021	0.49	0.622	−0.062, 0.103	0.751	1.33
Snapchat	0.056	0.042	0.056	1.32	0.189	−0.027, 0.139	0.712	1.40

Reference categories: female, arts faculty, first academic level, and non-use of each platform. Daily use was entered as an ordered intensity variable.

### 4.5 Multiple regression predicting Axis 2

The full model predicting Axis 2 was statistically significant, R = 0.659, R^2^ = 0.435, adjusted R^2^ = 0.412, F (15, 368) = 18.86, *p* < 0.001. Daily-use intensity again emerged as the strongest predictor (B = 0.198, β = 0.585, *p* < 0.001). Facebook use retained a smaller positive association (B = 0.095, β = 0.109, *p* = 0.026). Other predictors did not make statistically significant unique contributions in the full model (Table 5).

**Table 5:** Full multiple regression model predicting Axis 2.

Predictor	B	SE	β	t	*p*	95% CI for B	Tolerance	VIF
Daily use	0.198	0.026	0.585	7.70	<0.001	0.148, 0.249	0.266	3.76
Gender: male	−0.039	0.032	−0.049	−1.23	0.221	−0.101, 0.023	0.953	1.05
Age	−0.017	0.023	−0.030	−0.74	0.462	−0.061, 0.028	0.918	1.09
Faculty: law	−0.041	0.054	−0.038	−0.77	0.445	−0.147, 0.065	0.626	1.60
Faculty: science	−0.037	0.045	−0.044	−0.81	0.420	−0.126, 0.052	0.520	1.92
Faculty: commerce	−0.029	0.051	−0.029	−0.57	0.573	−0.130, 0.072	0.584	1.71
Faculty: other	−0.065	0.057	−0.055	−1.14	0.255	−0.176, 0.047	0.656	1.52
Level: second	−0.042	0.051	−0.046	−0.83	0.405	−0.142, 0.058	0.515	1.94
Level: third	0.004	0.052	0.004	0.07	0.943	−0.098, 0.105	0.525	1.90
Level: fourth or above	−0.030	0.047	−0.037	−0.64	0.522	−0.123, 0.062	0.454	2.20
WhatsApp	0.051	0.047	0.056	1.08	0.281	−0.042, 0.144	0.569	1.76
Facebook	0.095	0.042	0.109	2.24	0.026	0.011, 0.178	0.644	1.55
Instagram	−0.059	0.041	−0.074	−1.45	0.148	−0.139, 0.021	0.593	1.69
Twitter/X	0.006	0.042	0.007	0.15	0.885	−0.077, 0.090	0.751	1.33
Snapchat	0.006	0.043	0.007	0.15	0.880	−0.078, 0.091	0.712	1.40

Reference categories: female, arts faculty, first academic level, and non-use of each platform. Daily use was entered as an ordered intensity variable.

### 4.6 Regression diagnostics

To ensure the integrity of the regression models, a comprehensive suite of diagnostic tests was performed. Multicollinearity was assessed through tolerance and variance inflation factor (VIF) statistics; across predictors, VIF values ranged from 1.05 to 3.76, indicating that multicollinearity was not a serious concern. Tolerance values ranged from 0.266 to 0.953. Durbin-Watson values were approximately 2.08 in both models, supporting the independence of errors. Breusch-Pagan tests were not significant for Axis 1 (LM = 22.99, *p* = 0.084) or Axis 2 (LM = 16.39, *p* = 0.356), indicating no strong evidence of heteroscedasticity. Standardized residuals were within acceptable ranges (Axis 1: −1.98 to 2.29; Axis 2: −2.13 to 2.83), and maximum Cook’s distances were small (0.022 and 0.034). Normality tests were significant, which is common in large samples, but residual plots did not suggest severe violations that would invalidate the OLS models. These checks support the stability and interpretability of the reported regression coefficients.

## 5 Discussion

The findings answer the research questions cautiously and in line with the cross-sectional perception-based design. Students reported high endorsement of social media as a perceived arena for awareness, moderation, and intellectual security, and they also strongly endorsed the perceived institutional-media role in countering extremist discourse. These findings should be interpreted as students’ perceptions of possible protective and preventive roles, not as evidence that social media objectively reduces extremism or that media institutions are demonstrably effective.

H1 was supported: Axis 1 and Axis 2 were positively correlated. This indicates that students who more strongly perceived social media as a space for awareness and intellectual resilience also tended to support the role of media institutions in countering extremist discourse. The association suggests that students do not view individual awareness and institutional mediation as competing alternatives; rather, they appear to understand intellectual security as a relational process involving personal discernment and credible public communication.

H2 was supported in the full regression model. Daily-use intensity was the strongest predictor of Axis 1, even after gender, age, faculty, academic level, and specific platforms were controlled. This result suggests that students who spend more time in digital environments are more likely to perceive social media as a relevant arena for awareness and moderation. The finding is consistent with platform and digital-public-sphere scholarship showing that everyday exposure and repeated participation shape how users understand public issues (Thorson, 2020; Tufekci, 2017; van Dijck et al., 2018).

H3 received partial support. Daily-use intensity significantly predicted stronger endorsement of the institutional-media role in countering extremism, and Facebook use retained a smaller positive association. However, most demographic variables and other platform indicators were not significant in the full models. The platform-specific pattern should therefore be interpreted cautiously. Facebook may matter because it is more likely than closed messaging environments to host semi-public discussion, institutional pages, awareness campaigns, and shareable civic content. WhatsApp, by contrast, may be highly used for interpersonal communication without serving the same public-facing institutional function. This interpretation is consistent with platform-specific evidence suggesting that Facebook can operate as a semi-public arena where civic messages, awareness campaigns, ideological claims, and threat narratives circulate together (Amin, 2024; Ashour et al., 2026). The Facebook association should therefore not be read as evidence that the platform is inherently protective; rather, it suggests that students may perceive Facebook as more institutionally visible and publicly argumentative than closed or primarily interpersonal platforms.

The analyses also clarify the multicollinearity issue in the model specification. Daily use and platform count were highly correlated, indicating that students who spent more time on social media also tended to use more platforms. For this reason, platform count was not entered into the regression models with daily use. Instead, the models included specific platform indicators, and VIF values were within acceptable ranges. This approach addresses potential redundancy while retaining the substantive distinction between intensity of use and platform type.

At a broader level, the results support a conditional and sociologically grounded account of intellectual security in digital environments. Students appear to perceive intellectual security as connected to everyday digital routines, institutional communication, and critical awareness rather than to simple restriction of information. This interpretation aligns with media-literacy scholarship, which treats resilience as a capacity to evaluate, contextualize, and respond to contested information, and with radicalization research showing that ideological influence is often diffuse, iterative, and embedded in networked communication (Conway, 2017; Hollewell and Longpré, 2022; Livingstone, 2004; Wardle and Derakhshan, 2017).

The findings also align with counter-radicalization and CVE scholarship showing that prevention depends on credible communication, community-sensitive messaging, and educationally grounded resilience rather than reactive restriction alone (Amit et al., 2021; Rabasa et al., 2010; Sageman, 2008). In this sense, universities and media institutions should treat intellectual security as a participatory literacy project that strengthens students’ interpretive agency while preserving openness, debate, and freedom of thought.

### 5.1 Limitations

Several limitations should be acknowledged. First, the study was cross-sectional, so the results cannot establish temporal order or causality. Second, all substantive findings are based on self-report perceptions and may be influenced by social desirability or normative responding. Third, the dataset does not include direct measures of exposure to extremist content, algorithmic curation, platform feeds, institutional message quality, or actual online behavior. Fourth, the sample was drawn from one university, which limits wider generalization beyond a regional Egyptian university context.

Lastly, certain administrative metadata, including the exact number of distributed invitations, the response rate, and specific fieldwork start and end dates, were not preserved in the retained audit file. The final sample size provided an adequate case-to-predictor ratio for the planned multivariate analyses, but the non-probability single-university design limits statistical generalization. The study therefore prioritizes transparent reporting of the final validated dataset, the listwise data-cleaning protocol, and post-estimation diagnostic checks to support the credibility of the reported associations.

Additional measurement limitations should also be noted. Content validity was documented qualitatively through expert review, but individual expert ratings were not retained; therefore, no formal CVI statistics are reported. Exploratory factor analysis was conducted in the pilot validation sample, but confirmatory factor analysis in an independent sample was beyond the scope of the available data. Future research should use confirmatory measurement models, multi-site probability or stratified sampling where feasible, behavioral platform data, and direct measures of exposure to extremist and counter-extremist content.

## 6 Conclusion and recommendations

This study examined students’ perceptions of the role of social media in awareness-oriented intellectual security and their perceptions of the role of media institutions in countering extremist discourse. The analysis shows that students generally view social media as a meaningful environment for awareness-building and moderation, while also expecting media institutions to play an active role in credible preventive communication.

The findings do not show actual causal effects of social media. Instead, they show that perceived intellectual security is associated with daily digital engagement and, to a smaller extent, Facebook use. Universities, media institutions, and policymakers should therefore strengthen digital literacy, critical-awareness training, and platform-specific communication strategies. Preventive communication should be embedded in the platforms students actually use and should be adapted to platform affordances, audience practices, and the need for credible, non-coercive counter-narratives.

Future studies should extend the present work by using longitudinal designs, multi-university samples, confirmatory measurement models, and behavioral indicators of platform exposure. Such designs would make it possible to distinguish perceived usefulness from actual communication effectiveness and to evaluate which forms of digital intervention genuinely strengthen youth resilience against manipulative or extremist persuasion.

## Data Availability

The original contributions presented in the study are included in the article/Supplementary material, further inquiries can be directed to the corresponding author.
