# Supplementary material for: University students’ perceptions of social media use and intellectual security: a cross-sectional study in Egypt
Source: Front Sociol. 2026 Jul 23;11:1877494. doi: 10.3389/fsoc.2026.1877494 (PMC13441929; doi:10.3389/fsoc.2026.1877494)
Supplement: Supplementary file 1 [file Table_2.DOCX]

# Appendix A.

# Survey Instrument

**Table A1**
*Demographic and Platform-Use Items*

| **Variable** | **Response options** |
| --- | --- |
| Gender | Male; Female |
| Age | 17–20; 21–23; 24–26 |
| College | Arts; Law; Science; Commerce; Other |
| Year of study | First; Second; Third; Fourth or above |
| Average daily social media use | Less than 1 hour; About 1 hour; 1–3 hours; 4–6 hours; More than 6 hours |
| Social media platforms used regularly | WhatsApp; Facebook; Instagram; Twitter/X; Snapchat |

*Note.* Participants could select more than one option for the item on regularly used social media platforms.

**Table A2**
*Likert-Scale Survey Items*

| **No.** | **Item** | **Strongly disagree** | **Disagree** | **Neutral** | **Agree** | **Strongly agree** |
| --- | --- | --- | --- | --- | --- | --- |
|  | **Axis 1: The Role of Social Media in Enhancing Intellectual Security Among Youth** |  |  |  |  |  |
| 1 | Social media contributes to raising awareness of the dangers of extremist ideologies. | □ | □ | □ | □ | □ |
| 2 | I follow content that promotes national values and intellectual moderation on social media. | □ | □ | □ | □ | □ |
| 3 | I realize that moderate use of social media enhances intellectual security. | □ | □ | □ | □ | □ |
| 4 | I can distinguish between moderate and extremist content on social media platforms. | □ | □ | □ | □ | □ |
| 5 | I believe intellectual awareness on social media is essential to protect youth. | □ | □ | □ | □ | □ |
| 6 | Social media affects my convictions and thoughts regarding public issues. | □ | □ | □ | □ | □ |
| 7 | The content spread on social media may change my stance on some intellectual matters. | □ | □ | □ | □ | □ |
| 8 | I interact with posts that reflect sound and moderate intellectual directions. | □ | □ | □ | □ | □ |
| 9 | I have noticed a clear influence of social media on the thinking of my friends and colleagues. | □ | □ | □ | □ | □ |
| 10 | I believe that social media plays a role in building or undermining intellectual security. | □ | □ | □ | □ | □ |
| 11 | Social media is effectively used to spread messages promoting intellectual moderation. | □ | □ | □ | □ | □ |
| 12 | I have noticed awareness campaigns on social media targeting youth. | □ | □ | □ | □ | □ |
| 13 | Visual content, including videos and images, on social media plays an effective role in intellectual awareness. | □ | □ | □ | □ | □ |
| 14 | I believe social media platforms can be used to combat extremist thinking. | □ | □ | □ | □ | □ |
| 15 | I follow accounts concerned with intellectual security and cultural awareness. | □ | □ | □ | □ | □ |
| 16 | Continuous use of social media helps develop my awareness. | □ | □ | □ | □ | □ |
| 17 | Balanced intellectual content on social media enhances intellectual security in society. | □ | □ | □ | □ | □ |
| 18 | Social media has guided me toward moderate thinking. | □ | □ | □ | □ | □ |
| 19 | Social media is an effective tool for correcting false intellectual concepts. | □ | □ | □ | □ | □ |
| 20 | Social media helped me understand the risks associated with intellectual extremism. | □ | □ | □ | □ | □ |
|  | **Axis 2: Utilizing Media to Counter Extremism and Support Intellectual Security** |  |  |  |  |  |
| 21 | Media institutions can utilize social media to combat extremism. | □ | □ | □ | □ | □ |
| 22 | Digital media can serve as a strategic tool to support intellectual security. | □ | □ | □ | □ | □ |
| 23 | Cooperation between media and awareness institutions enhances efforts to combat extremist thought. | □ | □ | □ | □ | □ |
| 24 | Media should work on correcting misconceptions spread through social media platforms. | □ | □ | □ | □ | □ |
| 25 | I believe that intellectual programs should be widely available on social media. | □ | □ | □ | □ | □ |

*Note.* All attitudinal items were rated on a five-point Likert scale: 1 = strongly disagree, 2 = disagree, 3 = neutral, 4 = agree, and 5 = strongly agree. Higher scores indicate stronger agreement with the perceived role of social media and media institutions in enhancing intellectual security and countering extremism.
